# European society for trauma and emergency surgery member-identified research priorities in emergency surgery: a roadmap for future clinical research opportunities

**DOI:** 10.1007/s00068-023-02441-3

**Published:** 2024-02-27

**Authors:** Gary Alan Bass, Lewis Jay Kaplan, Christine Gaarder, Raul Coimbra, Nathan John Klingensmith, Hayato Kurihara, Mauro Zago, Stefano Piero Bernardo Cioffi, Shahin Mohseni, Michael Sugrue, Matti Tolonen, Cristina Rey Valcarcel, Jonathan Tilsed, Frank Hildebrand, Ingo Marzi

**Affiliations:** 1 Division of Traumatology, Emergency Surgery and Surgical Critical Care, Perelman School of Medicine, University of Pennsylvania, 51 N. 39th Street, MOB 1, Suite 120, Philadelphia, PA 19104 USA; 2 Leonard Davis Institute of Health Economics (LDI), University of Pennsylvania, Philadelphia, PA USA; 3 Center for Perioperative Outcomes Research and Transformation (CPORT), University of Pennsylvania, Philadelphia, PA USA; 4 Surgical Critical Care, Corporal Michael J Crescenz VA Medical Center, 3900 Woodland Avenue, Philadelphia, PA 19104 USA; 5 Department of Traumatology at Oslo University Hospital Ullevål (OUH U), Olso, Norway; 6 Riverside University Health System Medical Center, Moreno Valley, CA USA; 7 Loma Linda University School of Medicine, Loma Linda, CA USA; 8 Comparative Effectiveness and Clinical Outcomes Research Center – CECORC, Moreno Valley, CA USA; 9 State University of Milan, Milan, Italy; 10 Emergency Surgery Unit, Ospedale Policlinico di Milano, Milan, Italy; 11 General & Emergency Surgery Division, A. Manzoni Hospital, ASST, Lecco, Lombardy Italy; 12 Sapienza University, Rome, Italy; 13 Department of Surgery, Sheikh Shakhbout Medical City (SSMC), Abu Dhabi, United Arab Emirates; 14 Division of Trauma and Emergency Surgery, Department of Surgery, Orebro University Hospital, 701 85 Orebro, Sweden; 15 Faculty of School of Medical Sciences, Orebro University, 702 81 Orebro, Sweden; 16 Letterkenny Hospital and Galway University, Letterkenny, Ireland; 17 Emergency Surgery, Meilahti Tower Hospital, HUS Helsinki University Hospital, Haartmaninkatu 4, PO Box 340, 00029 Helsinki, HUS Finland; 18 Hospital General Universitario Gregorio Marañón, Madrid (HGGM), Madrid, Spain; 19 Hull Royal Infirmary, Anlaby Road, Hu3 2Jz, Hull, England UK; 20 Department of Orthopaedics Trauma and Reconstructive Surgery, University Hospital RWTH Aachen, Aachen, Germany; 21 Department of Trauma, Hand and Reconstructive Surgery, University Hospital Frankfurt, Frankfurt, Germany

**Keywords:** Emergency Surgery, Research, Diagnosis, Treatment, Implementation Science, Delphi Technique

## Abstract

**Background:**

European Society for Trauma and Emergency Surgery (ESTES) is the European community of clinicians providing care to the injured and critically ill surgical patient. ESTES has several interlinked missions – (1) the promotion of optimal emergency surgical care through networked advocacy, (2) promulgation of relevant clinical cognitive and technical skills, and (3) the advancement of scientific inquiry that closes knowledge gaps, iteratively improves upon surgical and perioperative practice, and guides decision-making rooted in scientific evidence. Faced with multitudinous opportunities for clinical research, ESTES undertook an exercise to determine member priorities for surgical research in the short-to-medium term; these research priorities were presented to a panel of experts to inform a ‘road map’ narrative review which anchored these research priorities in the contemporary surgical literature.

**Methods:**

Individual ESTES members in active emergency surgery practice were polled as a representative sample of end-users and were asked to rank potential areas of future research according to their personal perceptions of priority. Using the modified eDelphi method, an invited panel of ESTES-associated experts in academic emergency surgery then crafted a narrative review highlighting potential research priorities for the Society.

**Results:**

Seventy-two responding ESTES members from 23 countries provided feedback to guide the modified eDelphi expert consensus narrative review. Experts then crafted evidence-based mini-reviews highlighting knowledge gaps and areas of interest for future clinical research in emergency surgery: timing of surgery, inter-hospital transfer, diagnostic imaging in emergency surgery, the role of minimally-invasive surgical techniques and Enhanced Recovery After Surgery (ERAS) protocols, patient-reported outcome measures, risk-stratification methods, disparities in access to care, geriatric outcomes, data registry and snapshot audit evaluations, emerging technologies interrogation, and the delivery and benchmarking of emergency surgical training.

**Conclusions:**

This manuscript presents the priorities for future clinical research in academic emergency surgery as determined by a sample of the membership of ESTES. While the precise basis for prioritization was not evident, it may be anchored in disease prevalence, controversy around aspects of current patient care, or indeed the identification of a knowledge gap. These expert-crafted evidence-based mini-reviews provide useful insights that may guide the direction of future academic emergency surgery research efforts.

**Supplementary Information:**

The online version contains supplementary material available at 10.1007/s00068-023-02441-3.

## Introduction

The evolution of surgical science is informed by six key and inter-related knowledge domains: 1) the translation of basic science discoveries to the bedside, 2) the aggregation of experiential learnings related to individual patient outcomes, 3) direct comparisons of techniques or surgical approaches (in the form of randomized controlled trials), 4) interrogation of large prospectively-accrued or retrospective administrative data sets, 5) synthesis of curated data as meta-analyses, systematic or narrative reviews, and 6) expert consensus opinions and guidelines. All these research methods are motivated by the desire to incrementally improve patient care.

Socioeconomic, logistic, or epidemiologic factors, as well as serendipity and opportunity, may influence the focus of surgical research. Recently, we have witnessed the overwhelming influence of the SARS-CoV-2 pandemic on large-scale population-based surgical observational research ventures with an almost-unprecedented and immediate change in clinical practice [1]. Initially, concerns regarding transmission of infection to operating team members during aerosol generating procedures as well as early associations with excess post-operative mortality for patients acutely infected with SARS-CoV-2 caused surgeons to pivot from established practice patterns to pursue either delayed operative management, or for certain conditions such as appendicitis, non-operative management [2–5]. With the post-pandemic return to relative normalcy, and the benefit of additional outcome data,, the standard-of-care appears to have safely tracked back towards practices established before the pandemic [6]. Accordingly, the clinical intersection of acute or chronic SARS-CoV-2 and emergency surgical pathologies management is de-emphasized in contemporary research inquiries.

In pursuit of enhanced patient outcomes, the European Society for Trauma and Emergency Surgery (ESTES), aims to address knowledge gaps in academic emergency surgery practice by identifying research priorities for collaborative research, patient-level data accrual in brief time bound prospective observational cohort studies, longitudinal outcomes registries, and research coordination. Potential avenues of emergency general surgery research relevant to ESTES members are presented within this manuscript.

## Methods

Using the modified eDelphi method [7, 8], an invited panel of ESTES-associated experts in academic emergency surgery generated a list of seven potential research priorities for the Society. Authors were jointly selected by the first, second, and last author based on several defining characteristics, including: prominence in the field of Emergency Surgery, service to the ESTES (including organization leadership Council service, as well as prior or current service within the organization), presentation history at national or international medical professional organization conferences, expertise in delivering or assessing education or educational methods, and relevant peer-reviewed manuscript publication history. The selection process strove to appropriately represent nation and gender diversity within the expert grouping.

**Fig. 1:**
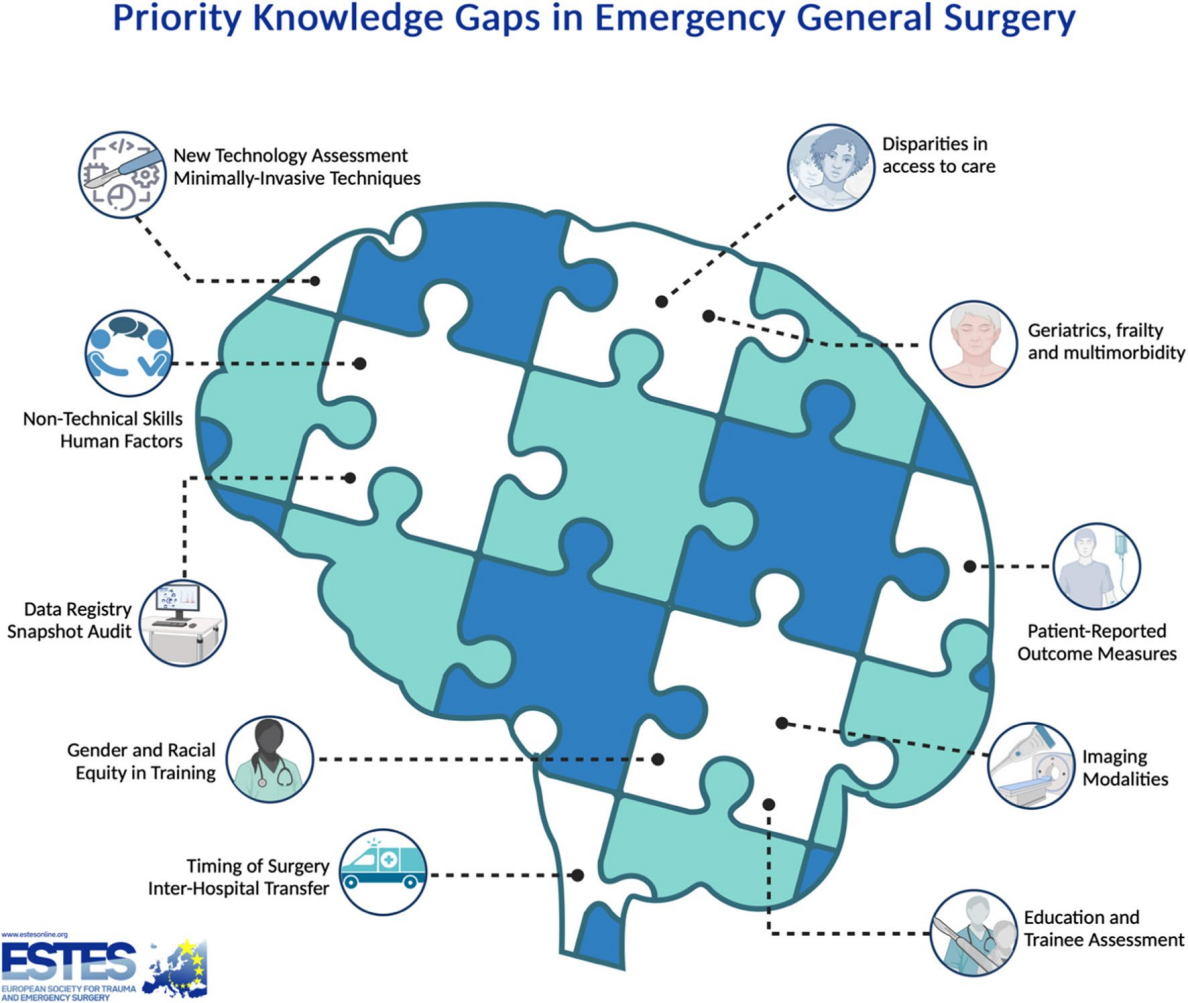
Potential knowledge and research gaps in Emergency General Surgery, identified by ESTES membership for future research prioritization: timing of surgery, pre-hospital care and inter-hospital transfer, diagnostic imaging in emergency surgery, the role of minimally-invasive surgical techniques and Enhanced Recovery After Surgery (ERAS) protocols, patient-reported outcome measures, risk-stratification methods, disparities in access to care, geriatric outcomes, data registry and snapshot audit evaluations, emerging technologies interrogation, and the delivery and benchmarking of emergency surgical training

Individual ESTES members with an emergency surgery practice were polled as a representative sample of end users and were asked to rank the potential areas of research according to their personal perceptions of priority. A REDCap® survey instrument [9] was delivered using an onscreen QR code link as part project presentation at the European Congress of Trauma and Emergency Surgery in Ljubljana, Slovenia (May 5th, 2023), and as a post-Congress electronic mailing from the ESTES administration to registered ESTES members. This survey instrument, which was anonymous and voluntary, asked respondents to prioritize the presented potential research topics on a Likert scale from highest (1) to lowest priority (7); data were subsequently binned at the analysis stage into high, medium, and low priorities. The following potential knowledge and research gaps were presented to the ESTES membership of ESTES for ranking regarding future research prioritization: 1) timing of surgery, 2) pre-hospital care and inter-hospital transfer, 3) diagnostic imaging in emergency surgery, 4) the role of minimally-invasive surgical techniques and Enhanced Recovery After Surgery (ERAS) protocols, 5) patient-reported outcome measures, 6) risk-stratification methods, 7) disparities in access to care, 8) geriatric outcomes,9) data registry and snapshot audit evaluations, 10) emerging technologies interrogation, and 11) the delivery and benchmarking of emergency surgical training (Fig. 1).

A free text field encouraged respondents to identify additional areas of priority that merited examination within this consensus statement. Respondents could voluntarily supply their name and the hospital where they worked if they were interested in pursuing future research projects; these data were used to aggregate responses by geographic practice region. The invited expert panel then synthesized survey results and framed their rationale behind why each domain was of key importance for emergency surgery research. Therefore, this consensus statement highlights knowledge gaps and potential essential avenues for academic emergency surgery research.

## Results

Seventy-two unique REDCap® responses were received; 56 (77.8%) respondents voluntarily provided their name, hospital, and country, while 16 (22.2%) respondents retained their anonymity. Of the 56 respondents who shared their identity, 39 (69.6%) were male, and 17 (30.4%) were female. Respondents flowed from twenty-three distinct countries and were combined into 7 regional groupings to simplify analysis (Supplemental Digital Content Table 1). Bar graphs describing the relative importance ascribed to each proposed research topic area are reproduced in full in the Supplemental Digital Content which accompanies this manuscript. Summary research priority themes are presented almongside examples of potential avenues of future inquiry (Table 1).

**Table 1:** Key take-home messages and targets for future investigation

Factors associated with outcomes in emergency surgery	Targets for future investigation
*Patient Transfer to higher level of care*	
While intuitively, one may conclude that transferring a patient to a higher level of care (higher performance hospital) is always accompanied by better outcomes, in emergency general surgery it may not be the case, as transfers may delay the timing of surgery.	Impact of transfers on emergency general surgery outcomes
*Timing of Surgery*	
In contrast to elective general surgery, an extensive assessment of chronic comorbidities as well as pre-operative optimization in patients sustaining emergency general surgery diseases, which would lead to delay in surgery in some cases, is not practical.	Impact of timing of surgery on outcomes of Emergency General Surgery patients.
*Surgical Procedure Risk*	
Emergency General Surgery encompasses a heterogeneous group of disease processes. It is imperative that to assess the two topics above, patients are stratified according to surgical procedure risk [10].	Analysis of ideal timing of surgery according to surgical procedure risk in EGS Is Failure-to Rescue associated with surgical procedure risk?
*Timing of Critical Interventions*	
Timing of advanced endoscopic and interventional radiology techniques may be lifesaving in several emergency general surgery conditions: Upper and Lower GI bleeding, Source control in abdominal sepsis, and biliary decompression in cases of calculous, acalculous, and infectious biliary obstruction. The availability, timing of intervention, and relevant outcomes should be evaluated using scientific methodology	Pre and Post outcomes comparison of the implementation of an advanced endoscopic intervention protocol for the diagnosis and treatment of upper and lower GI bleeding Pre and Post outcomes comparison of the implementation of an advanced endoscopic intervention protocol for the diagnosis and treatment of acute cholangitis Pre and Post outcomes comparison of the implementation of an advanced endoscopic intervention protocol for the diagnosis and treatment of obstructive jaundice Pre and Post outcomes comparison of the implementation of an advanced IR intervention protocol in the management of intra-abdominal abscesses. Pre and Post outcomes comparison of the implementation of an advanced IR intervention protocol in the management of GI bleeding of unknown origin.
*Emergency General Surgery Registries*	
The importance of clinical registries cannot be overemphasized. Well-designed EGS national registries with enough clinical granularity must be developed to serve two main purposes. Continuous quality improvement/performance improvement and research in EGS.	National effort coordination by ESTES and a European EGS registry could emerge.
*Imaging Modalities*	
The technological evolution of high-definition imaging associated with computerized imaging manipulation (e.g., 3-D imaging) allowed its widespread application in medicine in general and emergency surgery in particular. However simple techniques such as contrast studies using plain films are still useful in some circumstances. Ultrasonography requires either a specialized professional or intensive training for non-radiology physicians, whereas CT and MRI scanning require special environments, personnel, and equipment. Diagnostic Laparoscopy should also be included as an imaging modality commonly used in emergency general surgery cases when CT and US failed to provide a definitive diagnosis.	Comparison of accuracy, time to diagnosis, and resource utilization between US and CT scan in inflammatory acute abdominal diseases. Usefulness of diagnostic laparoscopy in patients with acute abdomen. Performance of contrast-enhanced CT (oral and iv) compared to plain film Gastrografin Challenge in the diagnosis of partial vs. complete small bowel obstruction.
*ERAS Protocols in Emergency General Surgery*	
Although pre-operative optimization is not feasible in Emergency General Surgery, several perioperative elements of the ERAS protocol should be implemented in all GES patients.	Defining the perioperative and postoperative elements of the ERAS protocol applicable to EGS procedures beyond exploratory laparotomy. Outcomes comparison between the rigorous use of perioperative and postoperative ERAS elements and standard of care in EGS patients.
*Minimally Invasive Surgery*	
Laparoscopic surgery has been broadly use in the management of several EGS disease processes. The advantages of a minimally invasive approach have been studied extensively and do not need to be repeated. Bailout strategies when a laparoscopic approach does not allow completion of the operation have not been studied as often. Additionally, several EGS programs have been using and reporting their outcomes after robotic surgery in the management of EGS diseases [11]	Laparoscopic surgery bailout approaches in difficult EGS cases: Is the open approach the only option? Comparison between the laparoscopic and robotic approaches in the management of specific General Surgical emergencies How to implement a robotic surgery program to manage EGS diseases? Defining a minimally invasive curriculum to train EGS surgeons.
*Patient-Related Outcome Measures, Quality of life, Palliative Medicine*	
	How do patient-reported outcome measures (PROMs) and QOL studies guide quality improvement activities and national benchmarking in EGS? How do patient-reported outcome measures (PROMs) and QOL studies guide individual patient care decisions in EGS? Effectiveness of Palliative Care Medicine in the management of EGS patients? When and How? Defining futility in EGS care.
*Disparities in EGS*	
An Assessment of racial, gender, and socio-economic disparities in EGS care.	How do disparities affect access to care, the type of surgical care received, and outcomes.
*Geriatric Emergency General Surgery Care*	
The interaction of multiple comorbidities, frailty, age, sex, and type of EGS disease process must be incorporated into models to determine modifiable risk factors associated with outcomes. More importantly, data is emerging suggesting that surgical procedure risk is more important than comorbidities and frailty as a risk factor for outcomes in elderly EGS patients. This is understandable since frailty is a non-modifiable factor as it relates to the timing and urgency or emergency of the surgical procedure in EGS diseases. These factors should all be considered in future research projects.	The impact of frailty on access to emergency surgical care, the type of surgical care received, and outcomes.

## Timing of surgery

Optimal patient-level surgical outcomes depend on several modifiable and non-modifiable factors, including structural aspects of where care is provided, resource constraints, and the technical and cognitive factors influencing the quality of surgical and perioperative care as well as care-sequencing [12]. While an association between survival and the achievement of composite quality measures like “the textbook outcome” [13] has been demonstrated in surgical oncology, a usable description of textbook outcomes in emergency surgery is largely absent and provides an opportunity for improvement. Exploring the optimal timing of surgery was aggregately assessed as a moderate research priority (Fig. 1)]. Beside the relevance of the correct time point for operative interventions in isolated illness [14], sequencing of care interventions is also of particular importance in patients with care rendered more complex by pre-existing multimorbidity [15]. Accordingly, point of care risk-stratification may provide opportunities to improve outcomes, especially by identifying those for whom surgical care may be inappropriate [16, 17].

Injury care generally does not provide time for risk stratification and instead uses damage control principles to minimize the potential morbidity of definitive surgical repair for those with hemodynamic instability or life-threatening hemorrhagic shock. Damage control principles include a staged approach based on acute hemorrhage control, control of soilage due to intestinal perforation, temporary fracture fixation, and may include debridement of devitalized wounds. After ICU resuscitation and stabilization, definitive treatment may be undertaken [14, 18, 19]. While well-utilized after injury, it remains unclear damage control is similarly advantageous for the non-injured critically-ill surgical patient (e.g. septic shock from feculent peritonitis, necrotizing soft tissue infection) and provides opportunities for future research [20, 21]

## Pre-hospital care and care prior to inter-hospital transfer

**Fig. 2:**
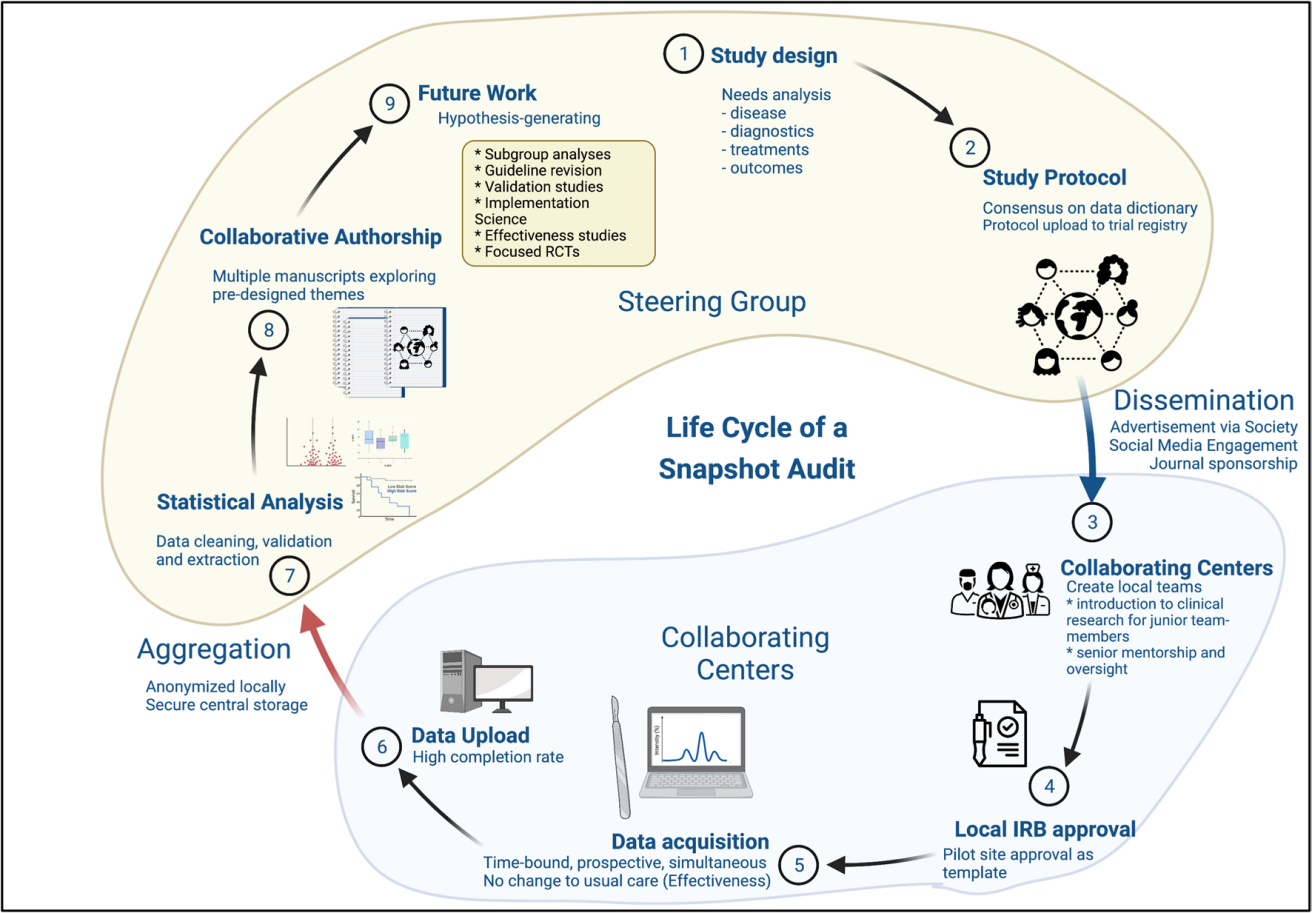
Snapshot audits conform to a similar structure: being time-bound, non-interventional, and multi-institutional. A successful diverse steering committee will leverage expertise that includes clinical care and data science, coupled with librarian services. Pre-published protocols (with specified aims and analyses) greatly aids site recruitment. Mentored trainee involvement at collaborating sites should be encouraged through manuscript contributorship. Current funding principally flows from medical professional organizations. The snapshot audit approach to assessing current care provides insights into care delivery, outcomes, and guideline compliance while generating testable hypotheses

These linked topics were aggregately scored as a medium priority area of future research, perhaps reflecting the presence of some data regarding regionalization of specific kinds of surgical care (Fig. 2). While a positive volume/outcome relationship has long been recognized in trauma surgery and surgical oncology following centralization of these services, emergency general surgical services remain widely offered, even in centers with low operative volumes. Relatedly, in many centers, after-hours emergency surgical care is provided by surgeons with predominantly elective practices. In Europe, there is no specific training program for emergency general surgery nor a formal acute care surgery model. Moreover, the effect of surgical hyper-specialization with the progressive erosion of traditional general surgery leads to progressively fewer surgeons with the skillset appropriate for complex emergency surgical care [22, 23]. In surgical oncology, patients cohorted in high-volume centers receive superior care leveraged on institutional familiarity and readiness. In contrast, patients suffering from critical surgical conditions requiring cognitive and technical skills unique to acute care surgery are generally transported to the closest available hospital as opposed to a specifically focused facility that centralized acute care surgery and the panoply of services that support such a program. Importantly, the effect of an integrated systems approach to the aftermath of acute care surgery—surgical critical care—is unexplored in the European context.

Inter-hospital transfer is inexorably connected with one of the central purposes of acute care surgery - rescue surgery. Failure to rescue (FTR), defined as the mortality rate after in-hospital complications (after elective or emergency general surgery or interventional radiology and endoscopic maneuvers), is used as a key-performance indicator to measure the ability of a hospital to identify and manage complications [24, 25]. To minimize the risk of death of critically ill patients needing a higher level of care, peripheral hospitals where patients’ needs overcome resource capabilities should be transferred to tertiary hospitals with lower FTR rate. A study of 200,000 Medicare patients undergoing six elective major surgical operations ranked different hospitals according to their rate of risk-adjusted mortality. Complication incidence was not statistically different between high performing vs. low-performing hospitals, but mortality was related to complication management (i.e. the ability for patient rescue) [26–28]. More recently, the most common complications requiring acute surgical rescue were surgical site complications, uncontrolled sepsis, and intestinal obstruction, resuscitation, critical care management, and source control using percutaneous or endoscopic approaches. Interestingly, 38% of cases were referred to the emergency general surgery service from other surgical services within the same institution, while an additional 26% were transferred from external institutions [29].

Insufficient data on EGS outcomes, due to the absence of large-scale patient-level EGS registries, make it difficult to identify patients a priori who are at greater risk of needing surgical rescue. Therefore, more intense efforts should be undertaken to develop EGS registries to better understand how patient-relevant elements interface with the complex systems involved in EGS care delivery [30].

Moreover, robust data collection should be routine in an ideal EGS system and will support Quality Improvement (QI) as well as an EGS standards verification process for Europe mirrored on the nascent American College of Surgeons Emergency General Surgery Verification Program (EGS-VP) [31–33], a new program paralleling the American College of Surgeons Committee on Trauma (ACS COT) trauma center verification approach [33]. In our current era of organ-specific or process specific surgeons interfacility transfers for EGS expertise have increased by approximately 150% in the United States over the past decade [34, 35]; there are no comparable contemporary data for Europe to inform policymakers. While coordinated inter-hospital transfer systems have been associated with a 9.7% decrease in EGS patient mortality, regionalization presents a variety of challenges, some of which may be related to finances, hospital systems, surgeon biases or desires, as well as patient preferences for specific care locations or care clinicians [24]

## Imaging modalities

Emergency general surgery patients – especially those who present with septic shock—require prompt diagnosis and management, and in this way, parallel the needs of injured patients for whom rapid imaging plays a key role in decision-making. To aid the acute care surgeon in developing the correct treatment strategy, a variety of imaging modalities exist, including portable radiography, point-of-care ultrasound (POCUS), computerized tomography (CT), and magnetic resonance imaging (MRI) [36, 37]. In high-stress situations, where quick decisions may be lifesaving, accurate real-time imaging is indispensable. By refining these techniques, technologies, and strategies for appropriate use, acute care surgeons can quickly obtain crucial information, leading to more timely interventions and improved patient outcomes. Unsurprisingly, this domain was rated as one with high priority in the aggregate.

Despite work to optimize the best strategies for imaging modality use in acutely ill surgical patients, there still exists areas for further research. Since emergency surgeons may also care for acutely injured individuals, it is appropriate to note potential opportunities synergistic body space imaging pathways that improve injury and non-injury care alike. Given the time-sensitive imperatives for injured patient management, developing imaging algorithms is critical. Many of the potential areas for improvement relate to the use of real-time imaging and the use and timing of CT, particularly in centers where CT imaging is readily accessible. Focused Assessment of Sonography in Trauma (FAST), being non-invasive and bedside-available, enables rapid and repeatable assessment of patients with abdominal injuries, particularly those with stab or gunshot wounds. While FAST was initially validated for evaluating blunt trauma patients [38], well-defined use and interpretation for those with penetrating abdominal injury remains uncertain [39]. Despite many centers routinely performing FAST – or its expanded version that assesses the thorax as well—for all injured patients, it is unclear the if benefit accrued by those with blunt injury is similarly realized by those with penetrating injury [40].

Surgeon-performed POCUS also provides many opportunities for the early bedside diagnosis of non-traumatic emergency surgical conditions [41, 42]. While the technique is encumbered by operator-dependent fidelity and requires a skilled and experienced sonographer to obtain accurate and reliable images, this limitation can be ameliorated through structured training [43, 44]. In emergency situations, where time is of the essence, the availability of a qualified operator might be limited, potentially delaying the diagnostic process. Additionally, bedside ultrasound may not always detect subtle findings, leading to potential false-negative results. In such cases, complementary imaging modalities like computed tomography (CT) are more sensitive but engender certain risks. CT remains more resource intensive and exposes the patient to a radiation source. More importantly, CT scanning is generally not portable (other than for intracranial imaging, extremity imaging, and some use for chest imaging during the pandemic) and requires the patient to leave the trauma bay [45]. With all these limitations, it places great emphasis on optimizing ultrasound use and algorithms for emergency surgical patients [46].

Emergency surgeons are often asked to evaluate and manage patients with suspected small bowel obstruction (SBO). A common evaluation tool is a water-soluble luminal contrast (Gastrograffin) challenge (GGC) that may be both diagnostic and therapeutic [47, 48]. The use of Gastrograffin can reduce the time to decision-making, expedite surgical intervention when indicated, and potentially decrease the overall length of hospital stay for patients with SBO [49, 50]. Despite the evidence supporting the GGC, the lack of a uniform administration procedure, standardized interpretation criteria, and action allows each center – and perhaps each surgeon – to implement unique practice management patterns [48]. A goal of pathway-based practice is to reduce individual variation to improve care quality. Given the potential for many different approaches for the same clinical condition identified by a GCC, this imaging modality seems ideal to characterize in detail and develop a consistent approach to utilization and interpretation.

## Minimally invasive surgery techniques

Minimally invasive surgery (MIS), coupled with Enhanced Recovery After Surgery (ERAS) pathways, is associated with faster post-operative recovery following elective surgery. As an extension of their elective practice, surgeons have increasingly adopted MIS into the management of surgical emergencies [51, 52]. For some emergency conditions, (e.g., acute cholecystitis) MIS (compared to open) is associated with fewer complications and shorter hospital stays; more complex conditions (perforated diverticulitis) may not enjoy the same complication and length of stay reduction. Whether adding an ERAS approach to MIS management of complex conditions would improve those outcome metrics remains to be delineated. Nonetheless, MIS approaches generally improve patient comfort. Accordingly, MIS techniques garnered a medium priority in the aggregate.

Both patient level outcomes and facility-relevant outcome metrics for emergency surgery are underexplored compared to elective surgical outcomes. While there are a variety of approaches to explore outcomes, some may demonstrate greater relevance to emergency surgery than others. Current inquiry highly values using prospective randomized controlled trials (RCTs) to assess therapeutics and are underpinned by a funding entity and substantial time and resource requirements. The inherent variability among emergency surgery patients can impede recruitment efforts as participants must meet rigid inclusion criteria. Nonetheless, RCTs comparing open and MIS approaches can be undertaken for certain emergency conditions such as adhesive small bowel obstruction and acute colonic emergencies each of which demonstrated that the MIS approach reduced recovery time [53, 54]. The above RCTS are more uncommon than other approaches to emergency surgery outcome inquiry. Given RCT logistical and methodological constraints, most EGS research has occurred as retrospective matched and non-matched studies documenting improved outcomes using a MIS approach [52, 55, 56].

When RCTs are not feasible, prospective observational and time-bound observational inquiries (known as snapshot audits) can assess the real-world management of intraoperative technical challenges and adverse events in a multinational fashion [57]. These studies would ideally focus on underexplored elements of EGS patient care including, but not limited to, triggers for conversion to an open procedure, complication frequency, time to restoration of gastrointestinal function, iatrogenic injury incidence and intra-operative recognition techniques, and what factors influence outcomes in those who require critical care. Regionalization for MIS in EGS patients may be also worthwhile to explore as outcomes may be related to surgeon preferences, skill sets and thereforebiases [11, 58]. Additionally, it is imperative to explore patient-reported outcomes (PROMs) during the in-hospital phase, as well as their long-term impact on post-convalescence quality-of-life.

## Patient-related outcome measures, quality of life, palliative medicine

The impact of surgical emergencies on patients' acute and long-term quality of life (QOL) remains under-investigated. Although emergency surgical care may have long-term physical and psychological effects less is known about the impact on their caregivers and related support structure(s). Understanding such sequelae can inform developing interventions to improve patient, caregiver, and support community-relevant long-term outcomes. Discovering what outcomes are most meaningful to patients – and how care has influenced those outcomes—is a point of embarkation in that process.

Patient-reported outcome measures (PROMs) have the potential to guide 1) individual patient care decisions, 2) quality improvement activities and national benchmarking, 3) payer mandates, and 4) population health research [59]. PROMs may consist of generic QOL measurements or condition-specific questionnaires [60]. Optimally, PROM collection would start before treatment and continue through convalescence – an approach that is best deployed in the elective setting as emergency care often precludes assessment prior to therapy. PROM collection should be straightforward to accommodate patients of varying capabilities, education, and socioeconomic strata; email survey collection has been demonstrated to be effective with excellent response rates in a UK study [61]. Electronic health record (EHR) data storage is ideal to facilitate analysis and subsequent use. Ideally, PROM data should be scored, risk-adjusted, and analyzed to enable time-based trend analyses as well as benchmark-based comparisons [59]. PROM analyses offer great potential to improve practice regardless of setting and remain underutilized in guiding outcome improvement. Accordingly, PROM analyses were scored as a medium priority research initiative.

## Disparities in access to emergency surgical care

Healthcare disparities, including those based on age, race, sex, and ethnicity, can have a significant impact on ability to access surgical care. Research has shown that racial and ethnic minorities often face barriers in accessing surgical care, including delays in diagnosis, limited access to specialized surgical services, and disparities in the availability or specific treatment options [62]. Recently, disparities in access to oncologic surgical interventions or solid organ transplantation or certain cancer approaches have been associated with poorer patient outcomes, including accelerated morbidity mortality [63, 64].

Understanding the root causes of healthcare disparities in surgical care and identifying the underlying social, economic, and cultural factors that contribute disparities is crucial. Research should focus on assessing the impact of unconscious biases, discriminatory practices, and structural inequalities within healthcare systems [65]. Efforts should be made to develop and implement interventions that can effectively reduce disparities in emergency surgical care such as targeted outreach programs, culturally competent care models, and policies that promote equitable access to surgical services. Despite such imperatives, respondents assigned this topic a medium priority, except in the Middle East where it was uniformly identified as a high priority.

## Data registries and prospective snapshot audit analyses

Data registries are essential for effective emergency surgery – not just emergency general surgery—collaborative research. The utility of large data sets in assessing the prevalence of specific conditions of interest, the penetrance of unique therapies, and real-world outcomes is highlighted by the interlinked approaches of data visualization and data science [66–68]. Therefore, the following priorities may be articulated: 1) development of a standardized data collection tool for acute surgical illness; 2) integration of data from multiple sources, including interoperable electronic health records, administrative databases, and patient-reported outcome registries; 3) establishment of a web-based platform for data collection, analysis, and dissemination using a common data dictionary [69]. Clearly many of these priorities cannot be accomplished without collaboration across national and medical professional organization boundaries. Coordination is necessary to avoid duplicative research efforts using divergent approaches. EGS patient population that represents 10% of global hospital admissions (exclusive or those related to military conflict). Comprehensive and accurate data curation are the foundations of focused inquiry and should be a priority examining global drivers of EGS care.

Accurate analysis and health system integration is exemplified by the Emergency Surgery Outcome Advancement Program eSOAP that helps drives reduced complication and mortality rates [70, 71], ESTES research strategy can lead the way. These priorities are relevant for ESTES considering its expanding research vision that targets life-saving interventions [30].

For some surgical conditions and scientific questions, the “real world” effectiveness of surgical patient care may be better explored using a multi-institutional time-bound observational cohort assessment approach (termed a “snapshot audit”) than by retrospective reviews of administrative datasets or by prospective randomized control trials (participation limited by inclusion criteria) (Fig. 2). Multi-center, ‘snapshot’. cohort studies or audits have the ability to gather large patient numbers in short time periods from a host of geographically diverse hospitals. Snapshot audits allow exploration of differences in patients, techniques, and management across the cohort to identify areas of practice variability that may result in putative outcomedifferences. As such, while not providing causal evidence of therapy efficacy or the impact of a particular intervention, sanpshot audits can be hypothesis-generating and may identify areas warranting further inquiry [72]. The European Society for Trauma and Emergency Surgery (ESTES) has recognized and actively embraces the strengths of this form of research, as well as its power in bringing together surgeons and emergency surgical units across multiple regions or countries for a common research goal, thus strengthening an active network of research participation across Europe. Nonetheless, this domains has been scored as a medium research priority except in Brazil and the Middle East where it was uniformly socred as a low priority.

## Geriatric surgical care – risk stratification and the interaction of age, frailty and multimorbidity

Advanced age, comorbidity bioburden, frailty, and urgent or emergent surgery are all independent predictors of post-operative complications including death [73, 74]. Indeed the impact of frailty on mortality is well preserved across different operative interventions, patient populations and settings [75]. Because there is a broad spectrum of morbidities, varying degrees of frailty, and different degrees of urgency for invasive care, a reliable and easy utilized approach to identifying the risk for specific complications as well as death is of great value for surgeons, patients, family members, or decision-makers.

Because many risk stratification tools were initially – and some exclusively – developed using elective surgical care populations, tools that are unique to those with advanced age, comorbidities, or frailty should be specifically developed. Furthermore, most risk stratification tools treat influencing conditions in a binary fashion (present/absent) but fails to account for interactions between included variables (e.g., heart failure with reduced ejection fraction and new onset KDIGO Stage 3 acute kidney injury) [17, 76].

To this end, studies that only includes elderly patients undergoing emergency surgery with specific interventions should be conducted to obtain population-based metrics for outcomes, and to support the development of novel tools for risk stratification. Machine learning/augmented intelligence are likely to prominently feature in such ventures. Specific care must be taken to ensure that the tools are usable with data that is available pre-operatively to help inform surgical decision-making including the decision to offer only non-operative care [77–80].

## New technologies to manage acute surgical disease

The introduction of new devices and their related use techniques is critical to the advancement of emergency surgical care, and outcome improvement. International guidelines, such as the IDEAL framework, provide a structured approach for implementation and evaluation of new surgical innovations [81, 82]. Despite the widespread availability of such guidelines, they have been poorly adopted, reinforcing the guideline impacting knowledge-to-practice gap that impedes practice change [83, 84]. Therefore, the incorporation of new technology into emergency surgical care has largely occurred in an unstructured fashion. Exemplar technologies include those current incorporate into practice including handheld ultrasound, endovascular intervention, and device placement tools, non- or less-invasive hemodynamic assessment tools, as well as a wealth of laparoscopic devices to facilitate MIS approaches. [85]

For example, endovascular revascularization techniques are quickly evolving driven by cardiac and neurological experiences with new thrombus fragmentation and suction devices, stents, and hybrid revascularization techniques [86]. Nevertheless, results including reduced mortality in comparison to open revascularization approaches are limited by retrospective analytic techniques, selection bias, and procedure heterogeneity [87–89]. Despite a lack of clarity regarding guidance for appropriate patient selection or adjunctive surgical techniques and their timing, endovascular treatment of acute mesenteric ischemia has been incorporated into expert consensus guidelines [90, 91]. Endovascular therapy is not the only novel technique that is increasingly deployed.

Indocyanine Green (ICG) imaging is another example of a promising new technique in emergency general surgery (EGS). Although it is not a novel technology, having received FDA approval in 1959, its current wide availability in our operating rooms and demonstrated safety, expanded its use [92]. In the emergency setting, fluorescence-guided surgery may improve duct visualization during laparoscopic cholecystectomy [93]. Intraoperative ICG angiography also provides a qualitative assessment of tissue perfusion and may support intraoperative decision–making in the evaluation of potentially ischemic [90, 94]. This technique is already widely adopted in elective minimally invasive colorectal surgery to evaluate anastomosis perfusion as some small RCTs and large ongoing studies appear to demonstrate a lower leak rate [95]; only animal models and case reports offer improved outcome data regarding bowel viability assessment after an ischemic insult. Because ICG is used across a wide array of indication and an patient types, deliberate analysis of its relationship to outcomes is warranted, especially for those with abnormal intestinal perfusion [96].

## Non-technical skills and human factor in EGS

There is increasing evidence that technical skills alone are insufficient for a successful surgical practice. Analysis of litigation records and closed-claims databases demonstrate that adverse events in surgical patients have been shown to result from poor decision-making and deficiencies in teamwork [97]. This realization has led to an increasing focus on non-technical skills (NTS) and human factors analysis to enhance patient safety and clinical outcomes. EGS constitutes a challenging setting for healthcare professionals regarding patient safety, due to disease complexity, burden heterogeneity, and the time-sensitive nature of EGS care. While some of these aspects are similar to injury care, NTS have been principally studied in the trauma settings, leaving the EGS setting an appropriate venue for exploration of NTS and team-based care opportunities [98, 99].

Validated NTS assessment tools for surgeons are structured around situational awareness, decision-making, communication skills, teamwork, and leadership [100, 101]. This framework provides a reliable assessment tool that may support the development of new training programs and evidence-based protocols to improve performance. The implementation of this framework in EGS may profoundly improve outcomes as emergency surgical patients have an eight-fold increased mortality compared to elective surgery patients, and up to 50% suffer postoperative complications [102].

Several studies have explored the need for NTS improvement through the entire perioperative patient journey including assessments of preoperative communication, shared decision-making, and effective conversations on goals of care and palliation [103]. The role of NTS in simulated operating theatre crises management has been examined as well, documenting NTS as essential for crisis management [104]. Relatedly, the benefit of utilizing a standardized communication tool to enhance OR-to-ICU handoff effectiveness and completeness also relies on NTS [105]. Nearly every aspect of EGS management offers a domain within which the role of NTS – and its training – may be examined. NTS inquiry clearly crosses professional boundaries and is likely to augment transactive learning and memory.

## Educational research into training, trainee assessment, case volume and variation between healthcare systems

Analyses of surgical training quality often focus on organizational and logistical issues of the training program and setting rather than the cognitive and technical as well as nontechnical aspects training [106–108]. Commonly, such explorations address tensions between trainee service commitment and activities believed to be beneficial for any aspect of training. In that context and following the reduction in work hours aimed at maintaining the health of the surgeons, providing an improved life-work-balance and enhancing patient safety, intensely examined elements include the impact of reduced exposure to clinical care as well as increased advanced practice provider (nurse practitioner or physician assistant) or attending-delivered care. Far less inquiry has addressed specific training methods.

Despite similar exposure to clinically relevant material or hand-on training, surgical trainees do not learn at the same rate [109]. Therefore, exposure alone is insufficient to ensure uniform training across groups of trainees in the same or different institutions let alone healthcare systems. Nonetheless, over the last two decades surgical training has slowly moved from traditional time-based models to ones that are competence-based. This shift requires the learner to master specific knowledge and skills as the metric for advancement. The former approach (time) is focused on the needs of the institution, while the latter (competence) addresses those of the trainee. The theoretical attraction of a competence-based curriculum is that it is more likely to produce more skilled surgeons and offers the potential to reduce total training time for rapid learners [110]. It is easier to provide greater responsibility and perhaps autonomy at an earlier stage of training for those with accelerated competency than it is to graduate such trainees more rapidly – care slots that are anticipated to be filled by those trainees would be left empty. Such an event is antithetical to patient safety and high-quality care. Planning for how to address such issues is a readily apparent research opportunity.

Methods advocated to implement competency-based training include improving work schedules to support educationally focused time, developing alternative approaches to initial skills acquisition (i.e., simulation trainers), shifting the service/training balance in favor of training, and improving the quality of the trainers to enhance training efficiency [111–115]. Unfortunately, many of these elements are devoid of data to support their efficacy. Other than Likert scale assessments of teachers by their learners, trainer *quality* is rarely defined and is often imprecisely assessed. For example, the Intercollegiate Surgical Curriculum Programme [https://www.iscp.ac.uk/] is promoted as a competence-based curriculum that was first developed in 2007 for the UK and Ireland and then deployed in England in 2018 as the Improving Surgical Training (IST) program [www.shapeoftraining.co.uk]. As a group, IST trainees, even in sites that did not fully comply with the IST program achieved higher standards in work-based assessments than those trained without the IST approach. They performed more procedures and progressed more rapidly in developing operative skills than their peers. However, it was not possible to determine whether these effects were due to the quality of training available at a site, the quality of supervision, the motivation and attitude of the candidate, or the specific relationship of the trainer with the trainee [114]. Moreover, in terms of global progression measured by all outcome categories recorded in their annual review of competency progression, IST trainees fared no better than their non-IST peers. Clearly, training enhancement analysis offers many domains on which scientific inquiry may focus, especially as they relate to emergency surgery training.

Furthermore, there is little data on how trainers should be trained for that role, how their efficacy should be objectively assessed, nor how they should be sustained. Relatedly, the relationship of trainer quality to training program credentialing remains unclear. There is a vast continuum of surgical volume, patient acuity, resources, and staffing across training programs with patient outcome excellence tied to all of those elements. It is intuitively attractive to also link each of those domains to training excellence, but such data are not necessarily clear, especially if most complex procedures are being performed by Attendings and Fellows, but not by surgical trainees. Currently, there are a few programs that help “train the trainer” but are medical professional organization specific and are not required by any credentialing body [116].

## Gender representation in emergency surgical training

Efforts to encourage women to train in trauma and emergency surgery are essential to increase diversity and gender equity in the field. Research has shown that women continue to be underrepresented in surgical specialties, including trauma and emergency surgery [117, 118]. To address this, it is important to promote mentorship programs and create supportive environments that empower women to pursue surgical careers. Increasing visibility and representation of successful female trauma surgeons through conferences, workshops, and medical professional societies can also inspire and encourage women to consider this field. Moreover, providing equal opportunities for training and career advancement, addressing implicit biases in selection processes, and promoting work-life balance initiatives are key steps towards attracting and retaining more women in trauma and emergency surgery. Furthermore, gender-based harassment whether personally directed or not, exerts a deleterious impact on field desirability – an influence that spans the entire breadth of medical training.

## Study limitations

The survey instrument presented to ESTES members listed expert-identified potential research priorities for surgeons engaged in emergency surgical research. Respondents were asked to rank the presented topics, without providing the rationale behind their prioritization. Thus, low priority might represent either a value judgment on behalf of the respondents that this was an area that they did not deem of sufficient research interest, or, conversely, that the literature was already either saturated, or there was sufficient evidence in existence that further research would not change clinical practice. The additional topics suggested by respondents were expanded by members of the expert panel but did not undergo member ranking. The examined priorities may be specific to those practicing in the European Union (EU) but included some members practicing outside of the EU. Therefore, the consensus statements and their underpinning data may not be equally applicable to other settings.

## Conclusions

This manuscript presents the priorities for ongoing research in academic general surgery as determined by a sample of the membership of the European Society for Trauma and Emergency Surgery. While this exercise was purely a ranking one which did not interrogate reasons for ascribing high medium or low priority to any area of research on an individual level, it provides useful insights that may guide the direction of future academic emergency surgery research efforts. Cross correlation of our results with a bibliometric analysis of the contemporary literature may provide further insights into whether the basis for prioritization was anchored in disease prevalence, controversy around aspects of current patient care, or indeed the identification of a knowledge gap. A list of statements and potential topics for investigation summarize the content of this article and may be used to provide direction and guidance for future research in Europe.

### Supplementary Information

Below is the link to the electronic supplementary material.

Supplementary file1 (DOCX 242 KB)
