# Publisher Correction: European society for trauma and emergency surgery member-identified research priorities in emergency surgery: a roadmap for future clinical research opportunities

**DOI:** 10.1007/s00068-024-02600-0

**Published:** 2024-08-22

**Authors:** Gary Alan Bass, Lewis Jay Kaplan, Christine Gaarder, Raul Coimbra, Nathan John Klingensmith, Hayato Kurihara, Mauro Zago, Stefano Piero Bernardo Cioffi, Shahin Mohseni, Michael Sugrue, Matti Tolonen, Cristina Rey Valcarcel, Jonathan Tilsed, Frank Hildebrand, Ingo Marzi

**Affiliations:** 1 Division of Traumatology, Emergency Surgery and Surgical Critical Care, Perelman School of Medicine, University of Pennsylvania, 51 N. 39th Street, MOB 1, Suite 120, Philadelphia, PA 19104 USA; 2 Leonard Davis Institute of Health Economics (LDI), University of Pennsylvania, Philadelphia, PA USA; 3 Center for Perioperative Outcomes Research and Transformation (CPORT), University of Pennsylvania, Philadelphia, PA USA; 4 Surgical Critical Care, Corporal Michael J Crescenz VA Medical Center, 3900 Woodland Avenue, Philadelphia, PA 19104 USA; 5 Department of Traumatology at, Oslo University Hospital Ullevål (OUH U), Olso, Norway; 6 Riverside University Health System Medical Center, Moreno Valley, CA USA; 7 Loma Linda University School of Medicine, Loma Linda, CA USA; 8 Comparative Effectiveness and Clinical Outcomes Research Center – CECORC, Moreno Valley, CA USA; 9 State University of Milan, Milan, Italy; 10 Emergency Surgery Unit, Ospedale Policlinico di Milano, Milan, Italy; 11 General & Emergency Surgery Division, A. Manzoni Hospital, ASST, Lecco, Lombardy Italy; 12 Sapienza University, Rome, Italy; 13 Department of Surgery, Sheikh Shakhbout Medical City (SSMC), Abu Dhabi, United Arab Emirates; 14 Division of Trauma and Emergency Surgery, Department of Surgery, Orebro University Hospital, 701 85 Orebro, Sweden; 15 Faculty of School of Medical Sciences, Orebro University, 702 81 Orebro, Sweden; 16 Letterkenny Hospital and Galway University, Letterkenny, Ireland; 17 Emergency Surgery, Meilahti Tower Hospital, HUS Helsinki University Hospital, Haartmaninkatu 4, PO Box 340, 00029 Helsinki, HUS Finland; 18 Hospital General Universitario Gregorio Marañón, Madrid (HGGM), Madrid, Spain; 19 Hull Royal Infirmary, Anlaby Road, Hull, England Hu3 2Jz UK; 20 Department of Orthopaedics Trauma and Reconstructive Surgery, University Hospital RWTH Aachen, Aachen, Germany; 21 Department of Trauma, Hand and Reconstructive Surgery, University Hospital Frankfurt, Frankfurt, Germany

**Publisher Correction**: **European Journal of Trauma and Emergency Surgery (2024) 50:367-382**

10.1007/s00068-023-02441-3

The original version of this article, published on 27 February 2024, unfortunately contained a mistake.

In this article Ingo Marzi should have been denoted as the second corresponding author.

The publisher apologizes for the error.

The original article has been corrected.

