# Supplementary material for: European society for trauma and emergency surgery member-identified research priorities in emergency surgery: a roadmap for future clinical research opportunities
Source: Eur J Trauma Emerg Surg. 2024 Feb 27;50(2):367–82. doi: 10.1007/s00068-023-02441-3 (PMC11035411; doi:10.1007/s00068-023-02441-3)
Supplement: Supplementary file 1 — Supplementary file1 (DOCX 242 KB) [file 68_2023_2441_MOESM1_ESM.docx]

Supplementary Digital Content

Table 1 - National and Regional declarations of survey respondents

|  | **Overall (N=58)** |
| --- | --- |
| **Country** |  |
| Spain | 10 (17.2%) |
| UK | 6 (10.3%) |
| Italy | 4 (6.9%) |
| Sweden | 5 (8.6%) |
| Finland | 3 (5.2%) |
| Norway | 3 (5.2%) |
| Belgium | 3 (5.2%) |
| USA | 3 (5.2%) |
| Denmark | 3 (5.2%) |
| Ireland | 3 (5.2%) |
| Switzerland | 2 (3.4%) |
| Brazil | 2 (3.4%) |
| Malta | 1 (1.7%) |
| Latvia | 1 (1.7%) |
| Romania | 1 (1.7%) |
| Czech Republic | 1 (1.7%) |
| Slovenia | 1 (1.7%) |
| Qatar | 1 (1.7%) |
| Netherlands | 1 (1.7%) |
| France | 1 (1.7%) |
| Poland | 1 (1.7%) |
| Cyprus | 1 (1.7%) |
| Oman | 1 (1.7%) |
| **Regional Grouping** |  |
| Nordic | 14 (24.1%) |
| Central/Western Europe | 14 (24.1%) |
| Spain | 10 (17.2%) |
| UK/Ireland | 9 (15.5%) |
| Italy | 4 (6.9%) |
| USA | 3 (5.2%) |
| Brazil | 2 (3.4%) |
| Middle East | 2 (3.4%) |


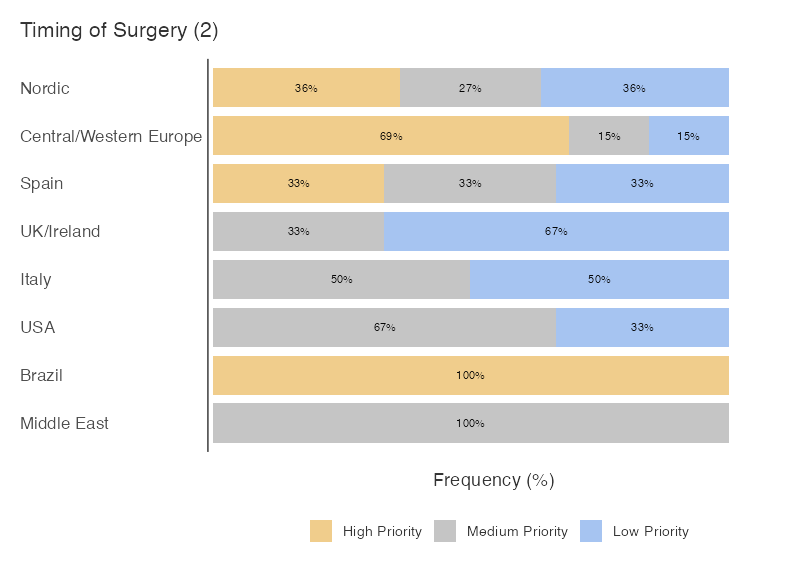


Figure 1 - Timing of Surgery. Survey responses, grouped by geographic practice location of respondents, and categorizeded into high-, medium- and low- priority for future research.


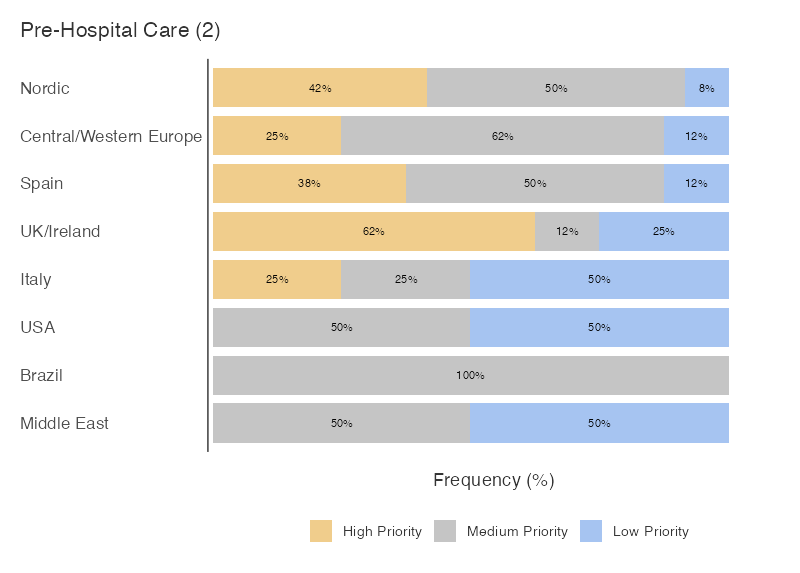


Figure 2 – Pre-hospital care and Inter-Hospital Transfer. Survey responses, grouped by geographic practice location of respondents, and categorizeded into high-, medium- and low- priority for future research.


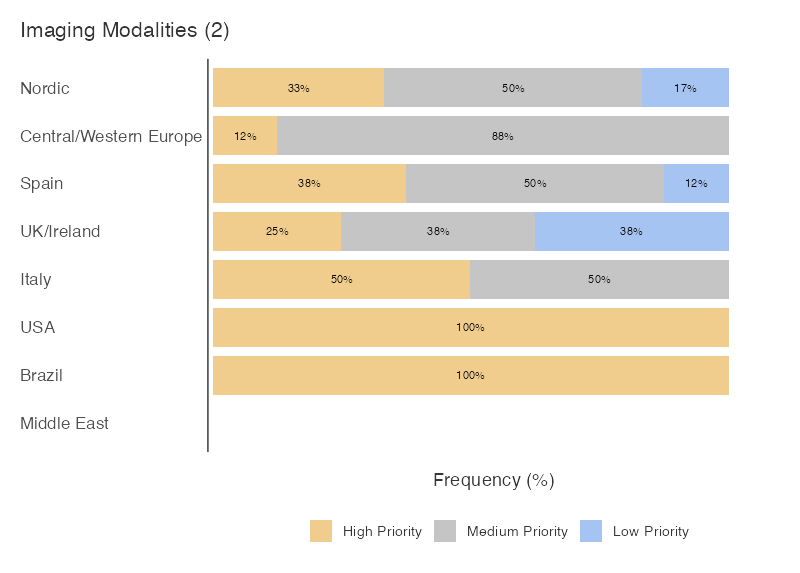


Figure 3 - Imaging in Surgial Decision Making. Survey responses, grouped by geographic practice location of respondents, and categorizeded into high-, medium- and low- priority for future research.


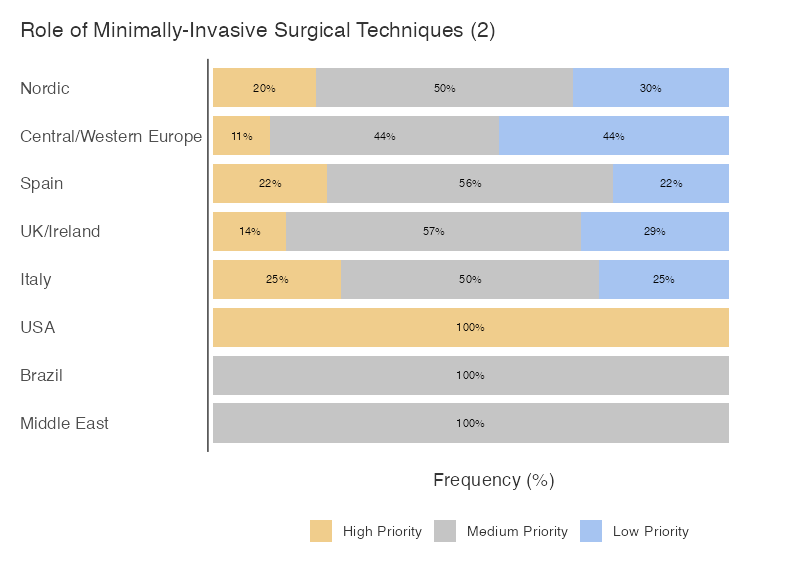


Figure 4 – Minimally-Invasive Surgery and new technologies. Survey responses, grouped by geographic practice location of respondents, and categorizeded into high-, medium- and low- priority for future research.


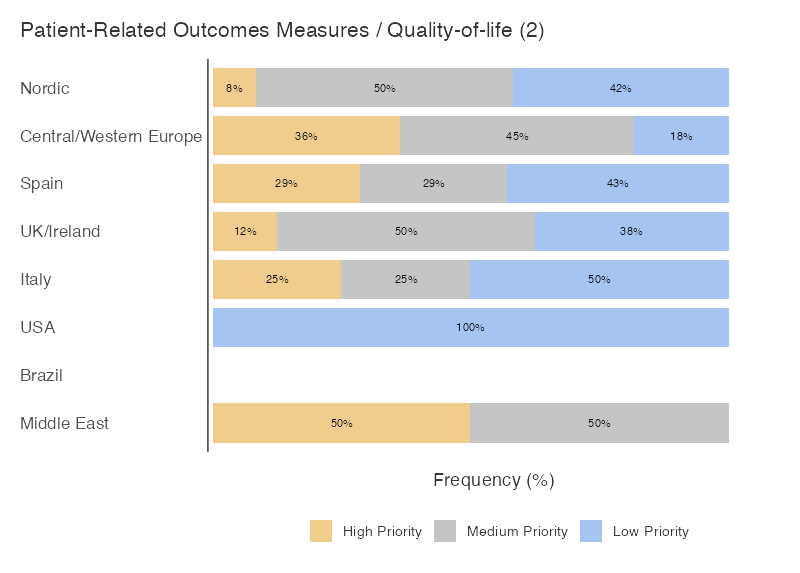


Figure 5 – Patient-reported Outcome Measures of Quality of Life.. Survey responses, grouped by geographic practice location of respondents, and categorizeded into high-, medium- and low- priority for future research.


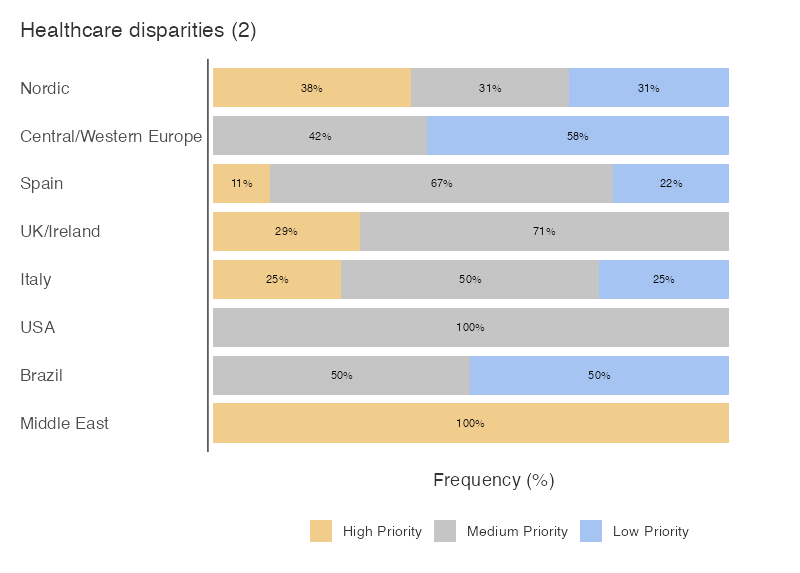


Figure 6 – Disparities in access to healthcare. Survey responses, grouped by geographic practice location of respondents, and categorizeded into high-, medium- and low- priority for future research.


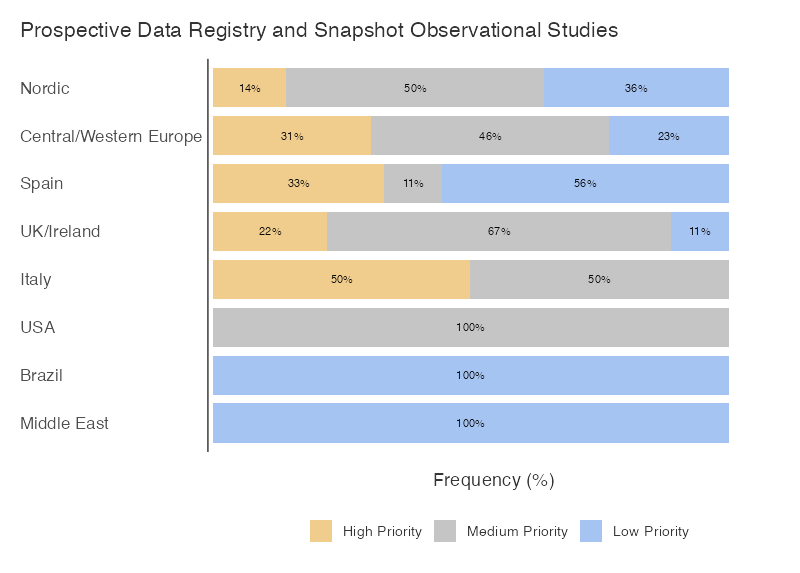


Figure 7 – Data Registry and Prospective Observational Cohort Studies. Survey responses, grouped by geographic practice location of respondents, and categorizeded into high-, medium- and low- priority for future research.
